# Seroprevalence of HTLV-½ in Macapá-AP, Brazil, in general population

**DOI:** 10.1590/0037-8682-0247-2025

**Published:** 2026-02-09

**Authors:** Amanda Lopes da Silva, Victor Ângelo Folgosi, Jorge Simão do Rosário Casseb, Márlisson Octávio da Silva Rego, Díuliana dos Santos Mendes, Júlia Pantoja Marques, Edcelha Soares D’Athaide Ribeiro, Antonio Charlys da Costa, Ester Cerdeira Sabino, Camila Malta Romano

**Affiliations:** 1 Universidade de São Paulo, Faculdade de Medicina, Laboratório de Virologia, São Paulo, SP, Brasil.; 2 Universidade de São Paulo, Faculdade de Medicina, Laboratório de Dermatologia e Imunodeficiências, São Paulo, SP, Brasil.; 3 Diretoria Executiva de Vigilância Laboratorial. LACEN/Superintendência de Vigilância em Saúde, Laboratório de Virologia, AP, Brasil.; 4 Universidade de São Paulo, Faculdade de Medicina, Hospital das Clínicas, São Paulo, SP, Brasil.

**Dear Prof. Dalmo Correia:**

Human T-cell lymphotropic virus type 1 (HTLV-1) was the first human retrovirus identified and isolated in 1980^1^. Since then, research on retroviruses has led to major advances, including the discovery of human immunodeficiency virus (HIV)^2^. However, HTLV remains a neglected infection despite its global distribution and potential to cause severe diseases such as HTLV-1-associated myelopathy/tropical spastic paraparesis (HAM/TSP)^3^ and adult T-cell leukemia/lymphoma (ATL)^4^. Infected individuals frequently present with inflammatory, dermatological, ocular, and rheumatological manifestations^5^. Vertical transmission, which is the primary route of infection, has remained unaddressed in Brazil for decades. In 2024, the Ministry of Health implemented nationwide HTLV-1/2 screening for pregnant women^6^ ^,^ ^7^, representing a critical step toward preventing new infections. 

In Brazil, as in many other developing countries, HTLV infections are often underreported due to a lack of awareness and inadequate screening. Vertical and sexual transmission, in particular, may go undetected, although the recent inclusion of testing in pregnant women may help in addressing this gap. However, HTLV is not routinely included in sexually transmitted infection (STI) screening at public health facilities, which may lead to an underestimation of cases among young adults who neither donated blood nor became pregnant.

Macapá, the capital of Amapá, lacks data concerning HTLV-1/2 seroprevalence in the general population. The most recent study (published in 2023) conducted in this region reported a seroprevalence of 0.2% in the state^8^. However, it included only blood donors who, despite their heterogeneity, do not reliably represent the true prevalence of HTLV-1/2 in the general population. In Brazil, most donors are repeat donors, predominantly young men (> 60%) with higher educational levels than the general population^9^. In the present study, we performed serological screening for HTLV-1/2 using immunoglobulin (Ig)M/IgG enzyme-linked immunosorbent assay (ELISA) (Wiener) of 740 samples from Macapá residents. Samples were conveniently obtained from individuals who presented to health centers with general symptoms of arboviruses in 2022 and 2023. 

The ELISA results were subsequently confirmed by western blotting (WB), which revealed an HTLV-1/2 seroprevalence of 0.27% (n = two samples), consistent with previously reported rates among Brazilian blood donors^8^ ^,^ ^9^. Notably, although seven samples tested positive by ELISA, only two were confirmed to be positive by WB and were therefore classified as true infections (Table 1). This finding underscores the critical role of confirmatory testing, particularly in low-prevalence populations, where the likelihood of false-positive ELISA results is high. Incorporating confirmatory tests enhances the reliability of seroepidemiological estimates.

**TABLE 1:** Overview of samples reactive for HTLV-1/2 in ELISA and western blot.

Sample	Sex	Ethnicity/Color	Age	Year of collection	ELISA	ELISA Cutoff	WB
1	M	Brown	22	2022	3,185	0,23	Positive
2	M	Brown	22	2022	0,285	0,23	Negative
3	F	Yellow	30	2022	0,231	0,22	Negative
4	M	Brown	36	2022	3,119	0,24	Positive
5	F	Brown	11	2023	0,264	0,24	Negative
6	M	Brown	12	2023	0,496	0,24	Negative
7	F	Brown	18	2023	0,551	0,24	Negative

**WB:** Western blot, **ELISA:** enzyme-linked immunosorbent assay.

In many Brazilian states, data regarding HTLV-1/2 seroprevalence are either unavailable or severely outdated. Aside from recent findings in Amapá, the most recent estimate was made in 2023 in Goiás, in the central-western region of the country and described a prevalence of 0.95%^10^. This higher positivity rate in Goiás likely reflects the demographic and behavioral profile of the population included in the study, which consisted mainly of Venezuelan refugees, including individuals from the Warao Indigenous group, where HTLV-2 infection is known to be endemic. 

Although HTLV-1/2 testing has been mandatory in blood donation centers since 1993^11^ and has recently been incorporated into prenatal screening programs in Brazil^7^, it remains absent from routine STI screening in primary health clinics. This omission highlights the persistent gap between national surveillance and prevention strategies. The lack of systematic testing in sexually active populations facilitates silent transmission, delays diagnosis, and increases the risk of secondary infections. Expanding HTLV-1/2 screening within existing STI programs would, therefore, represent a crucial step toward interrupting transmission chains and improving the early identification and management of infected individuals.
